# Attitude toward leisure, satisfaction with leisure policy, and happiness are mediated by satisfaction with leisure activities

**DOI:** 10.1038/s41598-022-16012-w

**Published:** 2022-07-09

**Authors:** Jieun Yoo

**Affiliations:** Department of Christian Education, Anyang University, 22, Samdeok-ro 37beon-gil, Manan-gu, Anyang-si, 14028 Gyeoggi-do Korea

**Keywords:** Psychology, Environmental social sciences

## Abstract

This study investigated the effect of attitude toward leisure and satisfaction with leisure policy on satisfaction with leisure activities and level of happiness in Korean caregivers for young children. Data were collected from 864 participants (397 men and 467 women) using the 2018 National Leisure Activity Survey (NLAS). The results showed that there were no significant differences between male and female caregivers in terms of satisfaction with leisure policy, satisfaction with leisure activities, and level of happiness. Women had more positive attitudes toward leisure. Satisfaction with leisure activities mediated the relationship between attitude toward leisure, satisfaction with leisure policy, and level of happiness; there were no significant differences between men and women with regard to the variables. The study suggests that attitude toward leisure and satisfaction with leisure policy promote the happiness of Korean caregivers for young children.

## Introduction

In the process of achieving economic development, South Korea has prioritized the sense of accomplishment that comes through work^1^; leisure activities used to be thought of only as ancillary means of improving labor productivity. Over the past decades, many Koreans have had negative attitudes about leisure activities that did not seem productive. Due to rapid economic development led by the government, incomes increased and the standard of living greatly improved, but Koreans’ satisfaction with life and their level of happiness remained low compared to other countries^2–4^. Most Koreans did not feel happy while engaging in leisure activities.

As Koreans have recently become increasingly interested in the topic of happiness, leisure has come into focus as a way of improving well-being^1,2^. Growth in the number of leisure-related studies has contributed to life satisfaction for individuals^5,6^ and families^7–9^, and the body of Korean literature related to the effects of leisure on life satisfaction is also growing^2,10,11^. However, previous leisure studies focused on participation in leisure activities and its effect on life satisfaction, and studies related to leisure attitudes and leisure policies were insufficient in number.

Leisure activities have a great impact on family health as well as personal happiness and life satisfaction of those who care for infants and toddlers^12^. With the knowledge that leisure is an important part of family caregivers' lives^13^, it seems appropriate to explore how the perception of leisure experiences and policies affect their leisure satisfactions. Although recent studies attempt to understand the parenting environment and psychological needs of parents through leisure, leisure-related studies on parents of young children are relatively scarce^14^. Additionally, most Korean researchers have indicated that participation in leisure activities has been associated with a higher quality of life among Korean adolescents^15^, college students^16^, and older adults^17^, but few researchers have studied caregivers for young children in Korea. Therefore, it would be meaningful to explore the relationships among attitude toward leisure, satisfaction with leisure policy, satisfaction with leisure activities, and happiness in Korean caregivers for young children.

### Attitude toward leisure

Veblen regarded leisure as a symbol of wealth and an activity different from work and/or unproductively spent time^18^. Similarly, some researchers have approached leisure (as opposed to work hours) as a time to pursue pleasure, engage in voluntary activities, and experience a feeling of freedom^19–21^. Others viewed leisure’s primary function as preparation for work, or even as compensation for the alienating effect of work^22^. Attitude toward leisure is defined as a willingness or predisposition to engage in leisure activities^23^. Attitudes toward leisure include beliefs, feelings, perceptions, knowledge, and behavioral components associated with leisure^24^. In Korea, attitude toward leisure is usually assessed based on individuals’ perceptions or beliefs about participating in leisure activities. These belief systems have changed because of social changes in Korea^1^. In the past, leisure was recognized and enjoyed as a subsidiary means to increase the production and efficiency of labor; however, leisure is now viewed as something that contributes to personal happiness and life satisfaction^1,25,26^.

One’s attitude toward leisure affects one’s satisfaction with one’s leisure time and activities. Some studies have shown that a positive attitude toward leisure is positively associated with a desire to participate in leisure activities^27^, which results in the development of satisfaction with leisure activities and time^28,29^. When an individual’s perceptions or emotions about leisure are positive, their satisfaction with leisure activities and time and with quality of life improve^30^. In addition, other Korean research supports the idea that a positive attitude toward leisure is positively correlated with satisfaction with leisure activities and time among college students^29,31^.

### Satisfaction with leisure policy

Leisure policy acknowledges the demand for leisure and systematically devises a plan to support leisure activities in accordance with individuals’ basic rights^1^. Leisure policies come from leisure-related government agencies that aim to increase people’s participation in leisure, create leisure spaces, administrate leisure policy processes, and secure leisure environments^32^. Since 1960, when overcoming national poverty was a top priority, Korea has pursued a labor-intensive production policy that relies on limited human resources. After growing as a labor-production-oriented society, Korea has established various leisure policies to reduce working hours and expand leisure time and activities to improve the quality of life for individuals^1^. For example, adopting policies such as five days or 52 hours of work per week and expanding the alternative holiday system increased individual leisure activities^33^.

Although environments where people can engage in leisure have improved and more public leisure facilities have been created through these policies, questions have arisen about the effect of these policies on the satisfaction that comes from leisure in Korea. The goal of the leisure policies was to improve the quality of life of individuals, but in practical terms, it was a supplier-oriented policy that did not take into account those who participate in leisure activities in Korea^1^. Therefore, the recognition and the evaluation of leisure policies have become important research topics as they relate to satisfaction with leisure activities. Previous studies related to leisure policy satisfaction have suggested the necessity of leisure policy evaluation through theoretical implications and examples^34,35^. Some Korean research found a positive relationship between satisfaction with leisure policy and satisfaction with leisure activities^1,36^, but related empirical studies are still scarce.

### Satisfaction with leisure and happiness

Satisfaction with leisure is understood as an individual’s positive evaluation of participating in leisure activities^27^. According to Beard and Ragheb, satisfaction with leisure is defined as positive personal perceptions or feelings derived from participating in leisure activities and resulting in the fulfillment of personal needs^37,38^. Happiness is considered essential to an individual’s life. According to the Greek philosopher Aristotle, a happy life is a virtuous life^39^. The term *happiness* is defined as “positive affect and subjective well-being”^40,41^ and is influenced by genetics, circumstances, and attitudes^42^. The concept of happiness is also related to life satisfaction or evaluation, subjective well-being, eudaimonia (i.e., psychological well-being), quality of life, or affect^43^.

A considerable amount of literature has shown a positive association between satisfaction with leisure activities and psychological well-being, life satisfaction, or happiness. According to Freire and Teixeira, leisure satisfaction has a direct effect on self-esteem, satisfaction with life, and psychological well-being^44^. Kaya found a positive association between satisfaction with leisure activities and happiness among college students^45^. Leisure satisfaction influences life satisfaction in Korean adolescents^46^. Wang et al.’s study showed, with regard to online games, a significant positive relationship between the physiological and aesthetic elements of leisure satisfaction and life satisfaction for adolescents^47^. All these studies provide empirical evidence that leisure satisfaction enhances happiness.

Recently, leisure-related research in non-Western countries has been increasing, but few studies have researched leisure in the context of caregivers in Korea. Leisure has had an impact on the lives of young children and their caregivers^48,49^. The happiness of caregivers affects children’s well-being and social and emotional development^50^. Leisure was found to play a role in ultimately improving the psychological satisfaction of parents and enhancing family health by preventing and/or alleviating stress^14^. Therefore, research on the relationship between leisure and happiness in Korean caregivers for young children is essential.

### Hypothesized model of attitude toward leisure, satisfaction with leisure policy, satisfaction with leisure activities, and happiness

**Figure 1:**
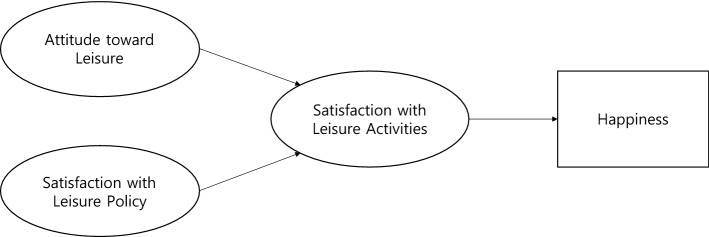
Path diagram of the hypothesized model.

Based on previous studies, it is plausible that one’s attitude toward leisure and satisfaction with leisure policy would lead to satisfaction with leisure activities and that satisfaction, in turn, would increase happiness. Therefore, this study aimed to investigate the hypothesized full mediational model in which attitude toward leisure, satisfaction with leisure policy, and happiness are fully mediated by satisfaction with leisure activities. Level of happiness has been reported differently by the different sexes. Subjective well-being was shown to be higher in women than in men, but the difference is not known to be significant^51^. Some Korean studies have shown gender differences in levels of happiness^52,53^. The present study sought to explore gender differences in the latent variables and the mediating pathway from attitude toward leisure and satisfaction with leisure policy to level of happiness, via satisfaction with leisure activities; structural equation modeling (SEM) with a nationally representative sample of Korean caregivers for young children (see Fig. 1), latent mean analysis (LMA), and multigroup structural model analysis were used for the purpose. The results would increase researchers’ understanding of the link between leisure and happiness and provide useful insights to improve the relationship between Korean caregivers and the young children in their care.

## Method

### Procedure

This study uses secondary data analysis from the 2018 National Leisure Activity Survey (NLAS), which collected data from a nationally representative sample of Korean individuals consisting of 10,496 participants over 15 years of age living in 17 cities and provinces. The NLAS involved a set of assessment modules designed by the Ministry of Culture, Sports, and Tourism to provide trend data reflecting changes in lifestyle and quality of life; the analysis reflects the status of leisure activities affecting demand for leisure according to various and changing domestic leisure environments. The NLAS consisted of a one-on-one interview by an investigator, and the survey period was from October 1, 2018, to November 30, 2018. The 2018 NLAS was performed under approval from Statistics Korea (Approval Number 113014). The raw data is publicly available from the Statistics Korea website (https://mdis.kostat.go.kr/index.do).

### Sample

For the current study, participants were selected from the respondents in the 2018 NLAS. The selected families consisted of caregivers (parents, mothers, fathers, grandparents, grandmothers, and grandfathers) aged 20–70 years with a preschool-aged child or children (3–6 years old). The sample included 864 participants (397 men and 467 women). The proportions of participants in their 20 s, 30 s, 40 s, 50 s, 60 s, and 70 s or older were 5.1%, 68.1%, 25.2%, 0.9%, 0.6%, and 0.1%, respectively. Of the total sample, 369 (42.7%) resided in metropolitan cities, 314 (36.3%) in medium-size cities, and 181 (20.9%) in rural areas. Participants’ education level varied as follows: 5 (0.6%) had completed middle school, 227 (26.3%) had completed high school, and 632 (73.1%) had a Bachelor’s degree or a graduate degree. They participated in six leisure activities—cultural and art-viewing activities, cultural and artistic participation activities, tourism, hobbies and entertainment, relaxation, and social and other activities (a category that included 87 kinds of leisure activities). The leisure types are presented in Table 1.

**Table 1:** Leisure types by participants.

Leisure type	Frequency	%
**Cultural and art-viewing activities**	18	2.1
Exhibition	1	0.1
Concert	2	0.2
Theater	4	0.5
Movie	11	1.3
**Cultural- and art-participation activities**	98	11.1
Musical instrument/singing class	3	0.3
Photoshoot	2	0.2
Indirect sports watching (TV or DMB)	12	1.4
Basketball, baseball, volleyball, soccer	2	0.2
Tennis, squash	1	0.1
Billiards	3	0.3
Bowling, table tennis	1	0.1
Golf	1	0.1
Swimming	5	0.6
Fitness exercise, aerobics	38	4.4
Yoga, Pilates	20	2.3
Badminton, jump rope	8	0.9
Tae kwon do, judo	2	0.2
Tourism	8	0.8
Visiting cultural heritage sites	1	0.1
Camping	3	0.3
Picnicking	1	0.1
Amusement park	2	0.2
Driving	1	0.1
**Hobbies and entertainment activities**	248	28.6
Crafts	3	0.3
Cooking	10	1.2
Pets	5	0.6
Climbing	1	0.1
Fishing	1	0.1
Internet surfing, SNS chatting	158	18.3
Internet games, PSP, PS3, Nintendo	47	5.4
Board game	2	0.2
Shopping	6	0.7
Drinking	4	0.5
Skin care, beauty-related activities	1	0.1
Reading books, watching cartoons	6	0.7
Learning languages	1	0.1
Gardening	3	0.3
**Relaxation activities**	437	50.6
Walking	32	3.7
Sauna	5	0.6
Napping	1	0.1
Watching TV (DMB, IPTV)	368	42.6
Watching videos (DVD, VOD)	5	0.6
Listening to radio (podcast)	4	0.5
Listening to music	20	2.3
Newspaper or magazine reading	1	0.1
Doing nothing	1	0.1
**Social and other activities**	45	5.2
Community service activities	1	0.1
Religious activities	5	0.6
Talking in person or on the phone	36	4.2
Dating	1	0.1
Meeting friends	2	0.2

Selected by allowing duplicates.

### Variables

#### Attitude toward leisure

Attitude toward leisure measured the perception of leisure, using two items. Participants were asked to respond to the following questions: “Do you think leisure activities are an essential requirement for life?” and ““Do you think leisure activities have a positive effect on your life?” on a scale of 1 to 7 (1 = strongly disagree, 7 = strongly agree). The Cronbach’s alpha coefficient for attitude toward leisure was 0.841.

#### Satisfaction with leisure policy

Satisfaction with leisure policy measured the satisfaction with the legal system and the infrastructure for leisure, using two items. Participants were asked to respond to the following questions: “Are you satisfied with the legal system related to leisure activities?” and “Are you satisfied with the leisure infrastructure?” on a scale of 1 to 7 (1 = very dissatisfied, 7 = very satisfied). The Cronbach’s alpha coefficient for satisfaction with leisure policy was 0.881.

#### Satisfaction with leisure activities

Satisfaction with leisure activities was measured using two items. Participants were asked to respond to the statements, “Are you satisfied with your overall leisure life?” and “Are you satisfied with the leisure activities you have participated in most in the past year?” on a scale of 1 to 7 (1 = very dissatisfied to 7 = very satisfied). The reliability coefficient, using Cronbach’s alpha for leisure satisfaction was 0.734.

#### Happiness

Happiness was measured using a single item. Participants were asked to respond to the question, “How happy do you think you are now?” on a scale of 1 to 10 (1 = very unhappy, 10 = very happy).

### Overview of analysis

For this study analysis, PASW 18.0 was used for the general characteristics of the participants and leisure activities, reliability, and correlation analysis of the measurement instruments. AMOS 18.0 was used for SEM to provide the hypothesized structural relationships among latent variables. This study evaluated the full mediation model of the relationship between attitude toward leisure, satisfaction with leisure policy, satisfaction with leisure activities, and happiness. In addition, this study conducted LMA and multigroup structural analysis to find differences between male and female groups^54^. While t-tests, ANOVA (analysis of variance), and MANOVA (multivariate analysis of variance) are typical methods for testing mean differences, LMA has the advantage of verifying the significance of mean differences between groups in consideration of measurement error^55^.  LMA can estimate the latent mean for males; the latent mean for females was set at 0. For LMA, invariance tests (e.g., configural invariance, metric invariance, and scalar invariance) were conducted in the hierarchical ordering of nested models. After the assessment of LMA was complete, a multigroup structural analysis was performed. The model fit with the maximum likelihood (ML) method and was evaluated using two relative-model fit criteria (Tucker-Lewis index [TLI] and comparative fit index [CFI]), one absolute model fit criterion (root mean-square error of approximation [RMSEA]), and the chi-squared difference test (△χ^2^ test).

### Ethics approval and informed consent

Ethical review for this study were waived because the data is secondary data (Approval No. is 113014). Informed consent was obtained from all individual participants included in the study.

## Results

### Descriptive statistics

Descriptive statistics with mean, standard deviations, skewness, kurtosis, and correlations for all variables are shown in Table 2. Overall, attitude toward leisure and satisfaction with leisure policy showed a positive correlation with satisfaction with leisure activities in both male and female caregivers. Two variables associated with attitude toward leisure were not correlated with satisfaction with leisure policy variables, but satisfaction with leisure activities was correlated with happiness in both groups. All significant correlations were positive. This is consistent with previous studies showing that attitude toward leisure and satisfaction with leisure policy are positively correlated with satisfaction with leisure activities^1,28,29,31,36^, and that satisfaction with leisure activities is positively related to happiness^13,44–47^. All values for skewness and kurtosis were within the range of $\pm$ 2 and $\pm$ 4, respectively, for normal distribution^55,56^.

**Table 2:** Correlations, mean values, standard deviation, skewness, and kurtosis of variables.

Variables	1	2	3	4	5	6	7
Attitude toward Leisure 1	1	0.768**	–0.030	–0.035	0.128**	0.077	0.192**
Attitude toward Leisure 2	0.650**	1	–0.001	–0.022	0.086	0.161**	0.226**
Satisfaction with Leisure Policy 1	0.018	0.007	1	0.789**	0.194**	0.169**	0.068
Satisfaction with Leisure Policy 2	0.044	0.004	0.791**	1	0.169**	0.177**	0.094*
Satisfaction with Leisure Activities 1	0.091	0.083	0.173**	0.137**	1	0.112*	0.288**
Satisfaction with Leisure Activities 2	0.234**	0.234*	0.245**	0.239**	0.087	1	0.142**
Happiness	0.255**	0.295**	0.166**	0.211**	0.295**	0.164**	1
M(SD)	5.45 (0.88)	5.58 (0.85)	4.75 (0.98)	4.91 (0.92)	5.54 (0.94)	4.18 (1.20)	6.89 (1.42)
Skewness	−0.139	−0.140	−0.212	−0.362	−0.297	−0.253	−0.530
Kurtosis	0.025	−0.224	0.089	0.392	−0.042	−0.306	0.673

*p < 0.05, **p < 0.01. The upper triangular matrix represents the correlation values for the female group, and the lower triangular matrix represents the correlation values for the male group.

### Construct validity test and fitness test of the model

Convergent validity considers how closely measurement instruments are related to the other variables of one latent variable. Construct reliability (CR) and average variance extracted (AVE) were calculated to measure convergent validity^57,58^. The values of CR above 0.7 and AVE above 0.5 are acceptable. In this study, convergent validity was acceptable because the values of CR were 0.891–0.896, with the value for leisure satisfaction close to 0.7, and the AVE values were 0.577–0.869. Discriminant validity is also acceptable because the AVE values are greater than the shared variance (i.e., squared correlation)^57^. Values around 0.08 are considered acceptable for RMSEA, while values of 0.05 are considered good; values of 0.90 or greater are considered good for TLI and CFI^57,59^. Thus, the model fit in the current study was acceptable (female group: χ^2^ (df = 11, N = 467) = 29.736, p = 0.002, TLI = 0.963, CFI = 0.981, RMSEA = 0.060, male group: χ^2^ (df = 11, N = 397) = 28.832, p = 0.002, TLI = 0.954, CFI = 0.976, RMSEA = 0.064).

### Construct equivalence test

All measurement models for assessing the three latent variables were compared to verify the configural invariance. Because the metric invariance model is an inherent model in the baseline model, it can be verified through the differences in χ^2^ using the difference in degrees of freedom between the two models. The fit of Model 1, the baseline model, shows high fit indices (see Table 3) (χ^2^ [df = 18, N = 864] = 41.795, *p* < 0.001, TLI = 0.968, CFI = 0.986, RMSEA = 0.039). To verify metric invariance, which has equal factor loadings on variables across groups, the χ^2^ values and the degree of freedom of Model 2, the metric invariance model, were compared against the baseline model, configural invariance. The difference between the χ^2^ values of the baseline model and the metric invariance model was not statistically significant at α = 0.05 (△χ^2^ [3, N = 864] = 2.856) (see Table 3); thus, metric invariance was established. TLI and RMSEA in the metric invariance model were accepted because the difference in the model fit was small (△TLI = 0.005, △RMSEA = -0.003). This result indicates that the measurement tool could be applied equally to both groups. Since the metric invariance model (Model 2) was established, the next step, scalar invariance, was evaluated. The scalar invariance model (Model 3) was not established because of a statistically significant result at α = 0.05 (△χ^2^ [6, N = 864] = 20.118). However, the fit indices for scalar invariance did not become worse than those for metric invariance (△TLI =  − 0.007, △RMSEA = 0.004), and the scalar invariance model (Model 3) was accepted^55^. The results showed that the measurement instruments and intercepts could apply equally to both groups. The observed mean differences in this study could reflect the differences between the groups for the latent variables.

**Table 3:** Fit indices for invariance tests.

Model	χ^2^	df	p	TLI	CFI	RMSEA
Configural invariance (baseline model): Model 1	41.795	18	0.001	0.968	0.986	0.039
Metric invariance: Model 2	44.651	21	0.002	0.973	0.986	0.036
Metric and scalar invariance: Model 3	64.769	27	0.000	0.966	0.978	0.040
Metric, scalar and factor variance invariance: Model 4	79.606	30	0.000	0.960	0.971	0.044

### Latent mean analysis (LMA)

Since configural, metric, and scalar invariance assumptions were all verified, LMA was carried out to see gender differences across the three latent variables (i.e., attitude toward leisure, satisfaction with leisure policy, and satisfaction with leisure activities) (see Table 4). The homogeneity of variance assumption was supported (△χ^2^ [3, N = 864] = 14.837, *p* < 0.001, △TLI = −0.006, △RMSEA = 0.004) according to the TLI, RMSEA, and χ^2^ difference tests comparing Models 3 and 4 (see Table 3). Thus, the *d* values were calculated using common standard deviations (see Table 4). Differences in attitudes toward leisure were defined as not small (*d* = 0.393) based on Cohen’s guidelines^54,60^. The results of the t-test showed no significant difference in level of happiness, an observed variable, between the female and male groups (t = -0.201, p = 0.841).

**Table 4:** Results of latent mean analysis.

Latent variables	Female (n = 467)		Male (n = 397)		Effect size	Total M
	Latent M	M	Latent M	M		
Attitude toward Leisure	0	5.609	−0.195***	5.406	0.393	5.516
Satisfaction with Leisure Policy	0	4.830	−0.004	4.826	0.006	4.828
Satisfaction with Leisure Activities	0	4.827	−0.060	4.900	0.007	4.859
Happiness	n.a	6.900	n.a	6.880	0.001	6.890

The latent mean values for females were fixed to zero. ***p < 0.001.

### Multigroup structural model analysis

**Figure 2:**
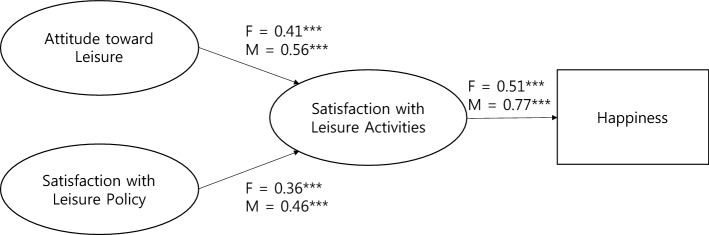
Model with equality constraints on factor loadings and path coefficients (standardized coefficients are shown for females and then males: ***p < 0.001).

In the multigroup structural model analysis, as a result of measuring the fitness of the path model, in which all factors for each latent variable were set the same way, the mediated model indicated a good fit with the sample data (χ^2^ [df = 25, N = 864] = 62.374, *p* < 0.001, TLI = 0.964, CFI = 0.978, RMSEA = 0.042). Table 5 presents the parameter estimates for both groups. To test the significant differences between path coefficients that might exist in the female and male groups, three models with equality constraints on the three path coefficients in the model were compared with the baseline model. The model fit remained almost unchanged, even if the equality constraint was applied to all path coefficients (χ^2^ [df = 42, N = 864] = 1754.106, *p* < 0.001, TLI = 0.964, CFI = 0.976, RMSEA = 0.041; △TLI = 0.000, △CFI = 0.002, △RMSEA = 0.001) (see Table 6). There were no statistically significant differences between the female and male groups in the equality constraints on each path. Attitude toward leisure and satisfaction with leisure policy had a strong influence on satisfaction with leisure activities; in turn, satisfaction with leisure activities had a strong influence on level of happiness for both groups. Figure 2 presents the model with equality constraints on path coefficients.

**Table 5:** Parameter estimates for female and male Groups (model with equality constraint on factor loading).

Parameter	Female	Male
Attitude toward Leisure → satisfaction with leisure activities	0.203***(0.405)	0.320***(0.564)
Satisfaction with leisure policy → Satisfaction with leisure activities	0.159***(0.355)	0.190***(0.459)
Leisure satisfaction → happiness	1.928***(0.505)	2.218***(0.767)

Parameter estimates are unstandardized coefficients. Standardized coefficients are given in parentheses. ***p < 0.001.

**Table 6:** Comparison of female and male group differences between the baseline model and models with equality constraints on the path coefficients.

Path with equality constraint on path coefficient	Δdf	Δχ^2^	*p*	ΔTLI
Attitude toward leisure → satisfaction with leisure activities	1	3.2	0.074	0.001
Satisfaction with leisure policy → satisfaction with leisure activities	1	0.305	0.581	−0.002
Satisfaction with leisure activities → Happiness	1	0.302	0.583	−0.002
All constrained	3	6.777	0.079	−0.001

## Discussion

While a substantial body of research on leisure has focused on the benefits of leisure activities among adolescents^30,46,61^, college students^29^, and adults^62^ in Korea, it is meaningful to examine the benefits of leisure for Korean caregivers of young children because they have less chance to engage in leisure activities while parenting in Korea. In addition, caregivers’ happiness plays a key role in children’s well-being^52^. Therefore, the current study aimed to explore how the effects of attitude toward leisure and satisfaction with leisure policy transferred to caregivers’ happiness via the mediation of satisfaction with leisure activities using SEM. The study also used LMA and multigroup structural analysis to explore gender differences associated with the variables and pathways.

The study results showed that a more positive attitude toward leisure and higher levels of satisfaction with leisure policy were associated with higher satisfaction with leisure activities, which was linked to higher levels of happiness. Consistent with previous research, the results support the idea that attitude toward leisure is positively related to satisfaction with leisure activities^28–31,63^, and satisfaction with leisure policy is positively correlated with satisfaction with leisure activities^1,36^. In addition, the association between satisfaction with leisure activities and level of happiness was consistent with previous research showing that greater satisfaction with leisure activities is related to greater life satisfaction and psychological well-being^28,44–47^. This finding suggests that caregivers for young children who had a more positive attitude toward leisure and greater satisfaction with leisure policy considered their satisfaction with their leisure activities to be beneficial for their happiness. This empirical evidence shows that satisfaction with leisure activities mediates the aspects of attitudes toward leisure and satisfaction with leisure policy that lead to greater levels of happiness. These findings are new, and they could reinforce the positive association between attitude toward leisure and satisfaction with leisure policy and greater happiness via satisfaction with leisure activities, especially for Korean caregivers for young children. Prior studies evaluated young adults (e.g., adolescents and college students), but this result uncovered a positive relationship in Korean family pairs. The results of this study, on the other hand, underline the important relationship between leisure experience (i.e., attitude toward leisure, satisfaction with leisure policy, and satisfaction with leisure activities) and level of happiness, for which there has been little research in Korea.

The LMA results supported the gender difference in only one variable: attitude toward leisure; the difference was not small, given the Cohen’s effect size (*d*-value = 0.393). In the multigroup structural analysis, attitude toward leisure and satisfaction with leisure policy had a significant indirect effect on level of happiness through the mediator of satisfaction with leisure activities for both groups; this outcome reflects full mediation. These findings indicated that female caregivers for young children had a more positive attitude toward leisure than male caregivers, but significant differences between path coefficients did not exist in either group. In general, women have more free time than men, but women’s participation in leisure activities is insufficient because of the burden of child care and familial duties. As a result, the quality of women’s leisure time is lower than that of men^64^. However, it can be assumed from the current study that the proportion of women participating in leisure activities is increasing because women’s household labor has been decreasing in Korean families. These findings indicate that, although Korean females are still subject to traditional gender roles in marriage^65^, their attitudes toward leisure can be positively enhanced.

In addition, the present findings underline the importance of satisfaction with leisure policy, as well as having a positive attitude toward leisure, for both female and male caregivers. In order to enhance an individual’s life satisfaction, not only is personal recognition of the importance of leisure time required, but also satisfaction with leisure policies in the legal system (e.g., paid parental leave or financial support for families with children) and leisure infrastructure. Such legislation and the establishment of leisure infrastructure are important to caregivers for young children because their satisfaction with leisure activities from government-led support tends to influence their children’s happiness. Therefore, leisure policy could be made to more effectively enhance the happiness of parents, especially when infrastructure, such as leisure facilities, programs, and professionals, is provided in tandem. It is believed that appropriate interventions for enhancing attitude toward leisure and satisfaction with leisure policy would help Korean caregivers perceive positive benefits from leisure activities and improve their assessments of their own happiness.

This study’s importance derives from the fact that it was the first to examine the relationships between attitude toward leisure, satisfaction with leisure policy, satisfaction with leisure activities, and level of happiness in Korean caregivers for young children. However, the study had some limitations. First, it focused on Korean caregivers for young children, but did not explore the multiple demographic factors might exist that influence their satisfaction with leisure activities, such as age, educational background, income, employment, etc. It might be interesting to investigate the factors that influence satisfaction with leisure activities among parent caregivers. The second limitation is related to the nature of the methodology used in this study. This cross-sectional study was designed to examine the psychological benefits gained by having a more positive attitude toward leisure and feeling greater satisfaction with leisure policy via satisfaction with leisure activities in Korean caregivers for young children. It would be beneficial for future research to qualitatively investigate the relationship between leisure experience and level of happiness among Korean caregivers for young children. Finally, this study did not differentiate between the types of Korean family structures that contain caregivers. There might be a difference in leisure activity tendencies among married parents, single parents, grandparents, etc. Future studies should investigate the relationships among the types of family structures, leisure, and happiness.

## Conclusion

This study aimed to explore the structural relationships among attitude toward leisure, satisfaction with leisure policy, satisfaction with leisure activities, and level of happiness in Korean caregivers for young children. Its results show that Korean caregivers’ attitudes toward leisure and satisfaction with leisure policy are important to their levels of happiness; satisfaction with leisure activities mediated the link between attitude toward leisure, satisfaction with leisure policy, and level of happiness, suggesting no gender difference. This study has advanced the literature on leisure by suggesting that satisfaction with leisure activities can promote happiness in Korean caregivers who are raising young children. The findings suggest that attitude toward leisure and satisfaction with leisure policy provide an avenue for improving satisfaction with leisure activities and facilitating happiness.

## Data Availability

Data are available upon request from the corresponding author. Data from this study originated from the National Leisure Activities Survey of Korea 2018.
